# Supplementary material for: Most Commonly-Consumed Food Items by Food Group, and by Province, in China: Implications for Diet Quality Monitoring
Source: Nutrients. 2022 Apr 22;14(9):1754. doi: 10.3390/nu14091754 (PMC9102302; doi:10.3390/nu14091754)
Supplement: Supplementary file 1 [file nutrients-14-01754-s001.zip › nutrients-1659304-supplementary.pdf]

Table S1. Percent consuming of DQQ sentinel foods in each food group compared to all items, nationally and by province (%).

Food groups	National	Beijing	Liaoning	Heilongjiang	Shanghai	Jiangsu	Shandong	Henan	Hubei	Hunan	Guangxi	Guizhou	Chongqing
Staple foods made from grains (group 1)	98.4	94.3	96.6	100.0	99.8	99.4	94.4	96.1	100.0	99.8	100.0	100.0	99.8
Whole grains (group 2)	98.9	96.2	98.7	100.0	96.6	100.0	99.6	100.0	100.0	100.0	100.0	88.7	100.0
White root/tubers (group 3)	96.7	95.1	98.7	99.7	96.2	91.3	98.7	99.1	96.9	88.6	83.1	99.2	96.5
Legumes (group 4)	98.1	95.0	98.9	99.7	92.2	99.3	99.5	99.1	99.3	100.0	97.3	99.2	99.1
Vitamin A-rich orange vegetables (group 5)	99.8	100.0	100.0	100.0	100.0	100.0	96.9	100.0	100.0	100.0	100.0	99.1	100.0
Dark green leafy vegetables (group 6)	99.2	96.8	98.5	99.5	99.6	99.3	100.0	98.7	99.0	98.7	99.2	100.0	100.0
Other vegetables (group 7)	97.5	98.4	99.7	99.6	98.1	99.3	99.2	98.9	99.2	97.4	90.5	93.1	97.6
Vitamin A-rich fruits (group 8)	96.5	89.7	80.0	96.0	100.0	100.0	96.8	100.0	100.0	97.1	95.7	100.0	100.0
Citrus (group 9)	100.0	100.0	100.0	100.0	100.0	100.0	100.0	100.0	100.0	100.0	100.0	100.0	100.0
Other fruits (group 10）	97.2	98.9	98.9	85.5	99.3	98.3	99.0	100.0	100.0	97.3	98.2	100.0	100.0
Baked sweets (group 11)	97.3	96.3	95.7	100.0	95.8	99.0	100.0	98.5	98.5	100.0	100.0	100.0	98.6
Other sweets (group 12)	99.3	95.8	100.0	100.0	100.0	100.0	100.0	100.0	100.0	100.0	100.0	100.0	100.0
Eggs (group 13)	100.0	100.0	100.0	100.0	100.0	100.0	100.0	100.0	100.0	100.0	100.0	100.0	100.0
Cheese (group 14)	100.0	100.0	NA	NA	100.0	100.0	NA	NA	NA	NA	NA	NA	NA
Yogurt (group 15)	100.0	100.0	100.0	100.0	100.0	100.0	100.0	100.0	100.0	100.0	100.0	100.0	100.0
Processed meats (group 16)	99.1	99.0	100.0	100.0	100.0	95.8	100.0	100.0	96.0	100.0	100.0	100.0	98.4
Unprocessed red meat (ruminant) (group 17)	99.5	99.4	100.0	100.0	100.0	100.0	100.0	98.3	100.0	99.0	98.4	100.0	100.0
Unprocessed red meat (non-ruminant) (group 18)	99.7	99.5	100.0	100.0	99.9	99.8	100.0	100.0	100.0	99.9	99.5	100.0	98.5
Poultry (group 19)	98.6	100.0	94.9	94.4	97.9	97.0	100.0	100.0	98.8	98.7	100.0	100.0	98.2
Fish and seafood (group 20)	100.0	100.0	100.0	100.0	100.0	100.0	100.0	100.0	100.0	100.0	100.0	100.0	100.0
Nuts and seeds (group 21)	95.0	92.0	94.8	94.2	95.2	81.2	97.6	98.1	98.0	98.3	94.9	100.0	98.9
Packaged ultra-processed salty snacks (group 22)	100.0	100.0	100.0	NA	100.0	NA	NA	NA	100.0	NA	NA	100.0	100.0
Instant noodles (group 23)	100.0	100.0	100.0	100.0	100.0	100.0	100.0	100.0	100.0	100.0	100.0	100.0	100.0
Deep fried foods (group 24)	99.0	99.2	100.0	100.0	98.1	95.2	100.0	100.0	95.3	100.0	100.0	100.0	100.0
Fluid milk (group 25)	100.0	100.0	100.0	100.0	100.0	100.0	100.0	100.0	100.0	100.0	100.0	100.0	100.0
Sweet tea/coffee/milk drinks (group 26)	100.0	100.0	100.0	100.0	100.0	100.0	100.0	100.0	100.0	100.0	100.0	100.0	100.0
Fruit juice (group 27)	100.0	100.0	100.0	100.0	100.0	100.0	100.0	100.0	100.0	100.0	100.0	100.0	100.0

SSBs (sodas) (group 28)	100.0	100.0	100.0	100.0	100.0	100.0	NA	100.0	100.0	100.0	100.0	100.0	100.0
Fast food (group 29)	100.0	100.0	100.0	NA	100.0	100.0	100.0	NA	NA	100.0	NA	NA	100.0

Note: SSB = sugar-sweetened beverages;

NA = not available;

Percentages between 90.0-95.0% are highlighted with a light shade, and percentages below 90.0% are highlighted with a dark shade.
